# New Uses for the Personal Glucose Meter: Detection of Nucleic Acid Biomarkers for Prostate Cancer Screening †

**DOI:** 10.3390/s20195514

**Published:** 2020-09-26

**Authors:** Clara Abardía-Serrano, Rebeca Miranda-Castro, Noemí de-los-Santos-Álvarez, María Jesús Lobo-Castañón

**Affiliations:** 1 Departamento de Química Física y Analítica, Universidad de Oviedo, Av. Julián Clavería 8, 33006 Oviedo, Spain; abardiaclara@uniovi.es (C.A.-S.); mirandarebeca@uniovi.es (R.M.-C.); santosnoemi@uniovi.es (N.d.-l.-S.-Á.); 2 Instituto de Investigación Sanitaria del Principado de Asturias, Avenida de Roma, 33011 Oviedo, Spain

**Keywords:** nucleic acid detection, prostate cancer, genoassay, personal glucose meter, alkaline phosphatase, point-of-care testing, handheld instrument

## Abstract

A personal glucose meter (PGM)-based method for quantitative detection of a urinary nucleic acid biomarker in prostate cancer screening, the so-called PCA3, is reported herein. A sandwich-type genoassay is conducted on magnetic beads to collect the target from the sample by specific hybridization, making the assay appropriate for PCA3 detection in biological fluids. The success of the method hinges on the use of alkaline phosphatase (ALP) to link the amount of nucleic acid biomarker to the generation of glucose. In particular, specifically attached ALP molecules hydrolyze D-glucose-1-phosphate into D-glucose, thus enabling the amplification of the recorded signal on the personal glucose meter. The developed genoassay exhibits good sensitivity (3.3 ± 0.2 mg glucose dL^−1^ pM^−1^) for PCA3, with a dynamic range of 5 to 100 pM and a quantification limit of 5 pM. Likewise, it facilitates point-of-care testing of nucleic acid biomarkers by using off-the-shelf PGM instead of complex instrumentation involved in traditional laboratory-based tests.

## 1. Introduction

Analyses at the point of need have aroused great interest in clinical diagnosis as well as in other areas such as food safety, environmental monitoring, or quality control due to their rapidity, portability, low cost, and easy use. In the case of healthcare, they are usually referred to as point-of-care or POC tests, which comprise diagnostic tests conducted out of the clinical laboratory and closer to the patient, by unskilled personnel or even the patients themselves [1]. These tests are generally designed for the detection of a certain target and they have to travel a long and expensive road before they are placed on the market, which limits the appearance of new devices for other targets.

There has been a recent trend to speed up this process, known as repurposing, based on expanding the capability of commercially available handheld devices to detect targets other than those intended. Thus, for example, simple devices used for monitoring physical or chemical parameters, such as pressure meters, thermometers, and pH meters, have been combined with biological recognition reactions enabling the determination of various types of clinical biomarkers [2,3,4,5]. Likewise, POC products already designed for the detection of biomarkers could be adapted for broadening the planned targets to other biomolecules of interest. Notable in this category are pregnancy tests, which detect the human chorionic gonadotropin. Conjugation of this hormone to a DNA oligonucleotide in combination with isothermal nucleic acid amplification techniques (mainly loop-mediated isothermal amplification or LAMP) has led to ultrasensitive detection of pathogens through their genetic material [6], although qualitative (yes/no) or semiquantitative results are typically obtained with lateral-flow dipsticks. Conversely, the personal glucose meter, PGM, is conceived for quantitative detection of glucose. This user-friendly device employed by millions of people with diabetes around the world is the result of many years of work in which the blood glucose meter evolved from photometric detection used in the 1960s until the current electrochemical detection using reactions based on oxidoreductase enzymes [7]. Considering its commercial availability at low cost as well as its portability and accuracy [8], the PGM has become a particularly promising portable meter for targets beyond glucose [9,10,11]. With this aim, different methods to correlate the concentration of the biomarker to the glucose signal recorded with the PGM have been reported, and they can be clustered into two main groups. The first one encompasses those methods based on encapsulation of glucose solutions in nanocontainers such as liposomes [12] and mesoporous particles [13], requiring a precisely controlled glucose release. The second group embraces those methods that involve enzyme-catalyzed glucose production. To this end, several enzymes have been reported in the literature, such as β-fructofuranosidase, also known as invertase, glucoamylase, and amyloglucosidase. Among them, the enzyme invertase stands out, especially when pursuing nucleic acid quantification [14]. This enzyme catalyzes the hydrolysis of sucrose into glucose and fructose. Temperatures around 55 °C are, however, required to achieve high enzymatic activity, thus demanding more complex equipment for glucose formation within a reasonable time. Moreover, invertase is marketed without modification, so cumbersome protocols involving heterobifunctional reagents are generally applied [15] for its conjugation to DNA probes. Furthermore, its large molecular weight (270 kDa) leads to a slower diffusion towards the sensing layer and, consequently, longer hybridization times. Thus motivated, we explored alternative glucose-generating enzymes that allow for overcoming the previous limitations associated with invertase, for quantification of a nucleic acid biomarker of prostate cancer using a glucose meter. Although prostate cancer screening has been traditionally tackled by monitoring prostate-specific antigen (PSA), a protein whose circulating levels increase in the serum of prostate cancer patients, its selectivity is reduced, leading to a high number of false positive results and, in turn, unnecessary biopsies and overtreatment [16,17]. An alternative biomarker for prostate cancer diagnosis able to discriminate between aggressive and indolent tumors is the nucleic acid prostate cancer antigen 3 (PCA3, also known as DD3). PCA3 is overexpressed (10–100 fold) in first-catch urine of prostate cancer patients after a digital rectal examination, and it exhibits higher selectivity and better predictive value than serum PSA [18]. Currently, the Progensa^®^ PCA3 assay offered by Hologic Inc (Marlborough, MA, USA) is the only PCA3 assay commercially available. This assay is based on an isothermal nucleic acid amplification of the biomarker combined with chemiluminescence detection, and its implementation requires the use of a bulky luminometer designed ad hoc [19]. In this work, we propose a simpler and cost-effective methodology for PCA3 determination which relies on the repurposing of the personal glucose meter, thus facilitating the screening of this condition.

## 2. Materials and Methods

### 2.1. Reagents

Streptavidin-coated magnetic beads (strep-MBs, 1 µm in diameter, 10 mg/mL, Dynabeads^TM^ MyOne^TM^ Streptavidin T1) were supplied by Thermo Fisher Scientific (Madrid, Spain). The enzyme conjugate streptavidin-beta-galactosidase (strep-β-Galac) was obtained from Thermo Fisher Scientific (Spain), while streptavidin-alkaline phosphatase conjugate (strep-ALP) and anti-Fluorescein-Fab fragment alkaline phosphatase conjugate (antiF-ALP) were from Sigma-Aldrich (Spain). The sugars D(+)-glucose, D(+)-lactose, D(+)-galactose, D(+)-glucose-1-phosphate, and D(+)-Glucose-6-phosphate, as well as the proteins streptavidin (strep) from *Streptomyces avidinii* and biotin labeled bovine albumin (BSA-biotin) were purchased from Sigma-Aldrich (Madrid, Spain) and used without further purification.

Magnesium chloride, 20× sodium-saline phosphate-EDTA solution (20× SSPE), 10× phosphate buffered saline solution (10× PBS), Tween 20 (70%), bovine serum albumin (BSA), biotin, and diethanolamine (DEA) were purchased from Sigma-Aldrich (Spain). Casein buffer (1%) in 1× PBS was acquired from Thermo Fisher Scientific (Spain).

Trinder reagent (a ready-to-use solution containing all components required for glucose determination by Trinder method including 0.4 mM 4-aminoantipyrine, 5 mM phenol, glucose oxidase (GOD) purified from *Aspergillus niger* > 10 U/mL and horseradish peroxidase (HRP) > 1 U/mL in phosphate buffer solution pH 7.5) were obtained from Biosystems (Barcelona, Spain).

DNA sequences (Table 1) involved in this work were synthesized by Metabion (Planegg, Germany), purified by HPLC, and provided in lyophilized format.

Ultrapure water (18.2 MΩ cm) was produced by a Milli-Q water purification system (Merck, Madrid, Spain) and it was employed in the preparation of all solutions.

All other reagents were of analytical grade and used as received.

Four different buffers were used: Washing buffer: SSPE-T (a 10-fold dilution from 20× SSPE supplemented with 0.01% Tween-20). Binding buffer: SSPE (same composition as SSPE-T except Tween-20). Blocking buffer (1× PBS-0.5% casein). Hydrolysis buffer whose composition depends on the glucose-generating enzyme.

### 2.2. Instrumentation

The glucose concentration was recorded with a GlucoMen^®^ AERO 2K personal glucose meter (A. Menarini Diagnostics, Barcelona, Spain) with a dynamic range of 20 to 600 mg/dL of glucose (1–33 mM). Glucose test strips incorporate glucose oxidase (GOD) as the enzyme employed to oxidize glucose and [Fe(CN)_6_]^3−^ as the redox mediator.

Incubation steps were conducted under gentle agitation in a Dynabeads^TM^ MX 12-tube Mixing Wheel (1.5 mL tubes containing ≥0.5 mL of solution were used with this vertical rotator), and magnetic separation was performed with a magnet incorporating a sample rack with 16 positions for standard 1.5 mL microcentrifuge tubes (DynaMag^TM^-2), both purchased from Thermo Fisher Scientific (Madrid, Spain). Temperature control during the enzymatic hydrolysis step was carried out in a ThermoMixer Comfort (Eppendorf, Madrid, Spain). Solutions were homogenized by shaking with an IKA^®^VORTEX Genious 3 (Merck, Madrid, Spain).

The spectrophotometric measurements (Trinder method) were performed with a GENESYS^TM^ 10S UV-Vis spectrophotometer (Thermo Scientific, Madrid, Spain), using disposable plastic cuvettes with an optical path length of 1 cm.

### 2.3. Sensing Layer Preparation of Magnetic Microbeads

Before surface modification of the microbeads, a buffer exchange is required to remove preservatives, namely sodium azide, present in the commercial stock solution. For this purpose, a 50-µL aliquot of the commercial suspension, previously mixed in a vortex, was transferred into a microcentrifuge tube and washed twice with (SSPE-T buffer) in a final volume of 1 mL. After each washing step, strep-MBs were magnetically entrapped on the wall of the tube in contact with a magnetic separator for 2 min, and the supernatant was discarded. The microbeads were then resuspended in 1 mL of SSPE buffer containing the biotinylated capture probe (1 µM, b-T_20_ or b-CP depending on the intended target). The mixture was incubated for 30 min in a vertical rotator at room temperature. Afterwards, the modified microbeads were separated from the mixture by magnetic attraction and washed twice with 1 mL of SSPE-T buffer to remove excess of b-T_20_ or b-CP. With the aim of blocking the remaining streptavidin binding sites on the captured probe-functionalized microbeads, these were incubated with 1 mL of a 50-µM biotin solution prepared in SSPE buffer for 30 min at RT in the vertical rotator. Excess biotin was subsequently washed off with SSPE-T buffer. After supernatant removal and reconstitution in 0.5 mL of SSPE buffer, modified microbeads were stored at 4 °C for further use.

### 2.4. Hybridization-Based Assay on Magnetic Microbeads

Quantification of the model target b-A_20_ was performed by means of a direct format genoassay, while the specific sequence of PCA3, T_PCA3_, was detected by using a sandwich-type assay involving two stages: a homogeneous hybridization between the target and the signaling and auxiliary probes, both in excess, followed by a heterogeneous hybridization between the ternary duplex preformed in solution and the surface-bound capture probe.

In both cases, 50 µL portions of the above captured probe-modified MBs were used. After 2 min of magnetic separation for removal of the supernatant, the MBs were resuspended in 500 µL of hybridization solution. Such a solution contains a variable concentration of b-A_20_ in SSPE, in the case of the model target; while for PCA3 detection, 250 µL of 500 nM signaling probe, 500 nM auxiliary probe, and variable concentrations of T_PCA3_ in SSPE buffer, previously subjected to a thermal shock (5 min at 98 °C for 5 min and gradual cooling until RT) to promote homogeneous hybridization, were mixed with 250 µL of SSPE buffer including 5% BSA. The mixture was incubated at RT for 2 h in the vertical rotator. After two washing steps with SSPE-T buffer to remove unbound DNA, the MBs were conditioned with the blocking buffer, which included casein to minimize nonspecific adsorption of the enzyme conjugate in the subsequent step.

Enzymatic labeling was then carried out by resuspending the MBs in 50 µL of blocking buffer containing the enzymatic conjugate (depending on the approach: 25 µg/mL strep-β-Galac, or 20 µg/mL strep-ALP, or 2 U/mL antiF-ALP) and mixing at RT. After 30 min of incubation time, the MBs were washed twice with SSPE-T buffer, once with SSPE buffer, and finally resuspended in 50 µL of hydrolysis buffer containing the glucose precursor enzyme substrate. For β-galactosidase enzyme, 50 µL of 100 mM lactose in 0.1 M phosphate buffer pH 7, including 1 mM MgCl_2_ was used. For alkaline phosphatase enzyme, 50 µL of 100 g/L D-glucose-1-phosphate in 0.1 M DEA pH 9.8, containing 1 mM MgCl_2_ was used.

Enzymatic hydrolysis reaction proceeded under temperature control (30 °C and 37 °C for β-galactosidase and ALP, respectively) and continuous stirring (1300 rpm) in a thermomixer, for a fixed time (1 and 2 h for β-galactosidase and ALP, respectively). Finally, the modified MBs were magnetically captured onto the wall of the microcentrifuge tube and the solution was collected for glucose quantification either with the PGM or with Trinder method. When working with ALP, the basic solution was previously neutralized by adding 2 µL of 2 M HCl.

### 2.5. Trinder Method

Trinder method is an enzymatic colorimetric method used for the determination of glucose [20]. The main enzyme is glucose oxidase, which catalyzes the oxidation of β-D-glucose into β-D-gluconolactone. For the colorimetric determination of glucose, a second enzyme, horseradish peroxidase, is incorporated, which reduces hydrogen peroxide as it is formed in the main enzymatic reaction, while generating a colored product (quinoneimine) with a maximum absorbance at 505 nm. The absorbance recorded at such a wavelength is directly proportional to the amount of dye produced and, in turn, to the amount of glucose initially present in the sample. This is a fixed-time spectrophotometric method in which the involved enzymatic reactions are left to proceed for a controlled time period, 5 min in this work, after which the absorbance is measured.

It was necessary to construct a calibration curve by using glucose standard solutions with concentrations ranging from 5 × 10^−5^ M to 5 × 10^−4^ M. Then, 800 µL of Trinder reagent was transferred into plastic cuvettes along with 200 µL of the corresponding standard solution (200 µL of water for the reagent blank), mixed and incubated for exactly 5 min at room temperature, and finally the absorbance of the mixture was measured at 505 nm. The calibration curve had to be obtained every day, just before the analysis of samples. An equivalent protocol was also followed for the determination of glucose generated by enzymatic hydrolysis in the genoassay. However, it may be convenient to dilute the solution with water in an appropriate proportion, in order to adapt the amount of glucose present to the linear response range of the colorimetric method.

### 2.6. Amplification Based on the Biotin-Streptavidin Recognition Event

The protocol to quantify the model target b-A_20_ through a direct genoassay, incorporating an additional amplification strategy, was as follows. First, 50 µL of MBs modified with the sensing layer was hybridized with the desired concentration of b-A_20_, as described in Section 2.4. Then, MBs functionalized with the biotinylated duplex were resuspended in 500 µL of a 30 µg/mL streptavidin solution prepared in SSPE buffer and kept at room temperature for 30 min under gentle stirring. After two washing steps with SSPE-T buffer and one with the blocking buffer, the MBs were then incubated with 500 µL of a 30 µg/mL BSA-biotin for 30 min in the vertical rotator. Afterwards, the MBs were washed twice with the blocking buffer to remove unbound BSA-biotin and subsequent steps of enzyme labeling, enzymatic hydrolysis, and glucose determination were performed as detailed in Section 2.4.

## 3. Results

**Figure 1:**
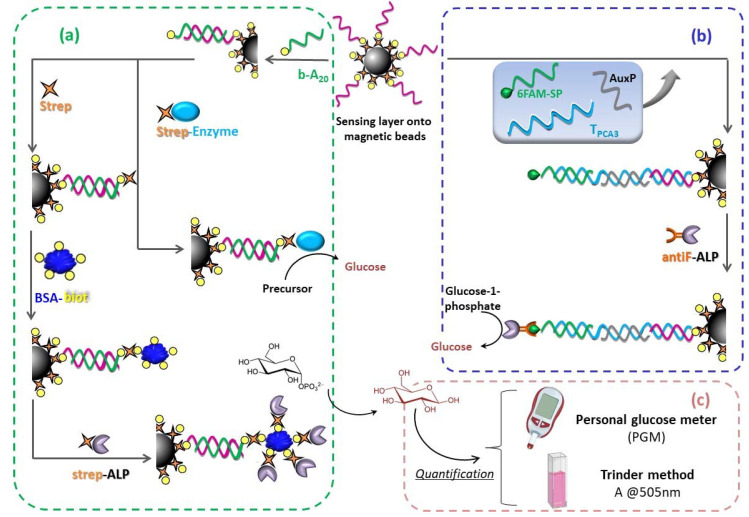
Schematic depiction of the hybridization-based bioassay. (**a**) Proof-of-concept direct genoassay for the detection of a model target b-A_20_; with/without amplification strategy based on multivalent system biotin-streptavidin. (**b**) Sandwich genoassay for the detection of T_PCA3_. Note that more than one capture probe is immobilized onto each MB but, for the sake of clarity, the figure only shows one DNA strand. (**c**) Quantification step, either with the personal glucose meter or spectrophotometrically.

In this work, we devise a method for the detection of a nucleic acid called PCA3, approved by the US Food and Drug Administration (FDA) as a urinary biomarker for prostate cancer diagnosis [21], in combination with a personal glucose meter, and magnetic particles as a solid support for a sandwich-type genoassay, as depicted in Figure 1b. With this purpose, an 85 mer synthetic DNA target of PCA3 RNA was designed by using the NCBI database. As previously discussed, one of the main strategies for detecting nucleic acid biomarkers with a personal glucose meter as the transducer consists of the employment of an enzyme that catalyzes the conversion of a glucose precursor substrate into glucose. Bearing in mind the shortcomings associated with invertase, we assessed alternative enzymes that lead to a reliable and feasible decentralized measurement device for prostate cancer screening.

### 3.1. β-Galactosidase

We initially evaluated β-galactosidase (β-Galac) as a glucose-generating enzyme alternative to invertase that, to the best of our knowledge, has not been assessed for such a purpose so far. β-Galactosidase catalyzes the hydrolysis of lactose into galactose and glucose, whose concentration could be detected with the PGM. This enzyme was supplied conjugated to streptavidin, which facilitates its binding to other molecules through the biotin-streptavidin affinity interaction. However, the stoichiometry of the enzyme conjugate (strep-β-Galac) is unknown as well as its source. Manufacturer does detail its specific activity and the recommended reaction buffer: 100 mM potassium phosphate pH 7.0 containing 1 mM MgCl_2_ and 0.1 M 2-mercaptoethanol. This latter thiol compound turned out to be incompatible with PGM readout, since signals superior to the upper limit of PGM were systematically recorded, even in the absence of glucose and, consequently, 2-mercaptoethanol was removed in the subsequent studies (see initial adjustments of PGM in Supplementary Materials).

According to the PGM instructions, galactose, the by-product generated during the hydrolysis of lactose, acts as an interference in glucose determination when present at concentrations greater than 15 mg/dL. However, in this particular case, it would be beneficial since it would contribute to increasing the signal recorded for a given glucose concentration, thus improving sensitivity. It was experimentally checked with standard solutions containing equimolar amounts of glucose and galactose, to simulate the solution resulting from enzymatic hydrolysis of lactose. A PGM signal enhancement of 16% with respect to the analogue solution containing only glucose was obtained (data not shown). Notice that the PGM used here is based on the enzyme glucose oxidase, which is more selective than glucose dehydrogenases.

In order to achieve optimal sensing performance, some important experimental parameters of the genoassay detection step were optimized, such as concentration of glucose precursor enzymatic substrate (lactose) and enzymatic hydrolysis time. These studies were performed with a model target b-A_20_ captured onto strep-MBs through hybridization with b-T_20_ by a direct format genoassay. To avoid error messages when glucose concentration is outside the response range of the PGM (20–600 mg/dL) as well as to save on test strips, a fixed-time enzymatic colorimetric method, known as Trinder method, was used in these optimization studies to determine the amount of glucose present in the solution. The protocol is detailed in Section 2.5.

To select the concentration of lactose to be used in the enzymatic hydrolysis step, a kinetic study of β-galactosidase-catalyzed production of glucose from lactose was carried out. To this aim, magnetic particles modified with the maximum amount of enzyme through hybridization event were prepared. Specifically, streptavidin-coated magnetic beads (strep-MBs) functionalized with the sensing layer, according to the protocol detailed in Section 2.3, were incubated with a 2 µM solution of b-A_20_ for subsequent labeling with strep-β-Galac enzyme conjugate. The enzyme-modified particles were dispersed in 50 µL of solutions containing increasing amounts of lactose, and after 30 min of incubation at 30 °C under gentle shaking, the solution was recovered by magnetic separation, and the amount of generated glucose was measured by the Trinder method.

**Figure 2:**
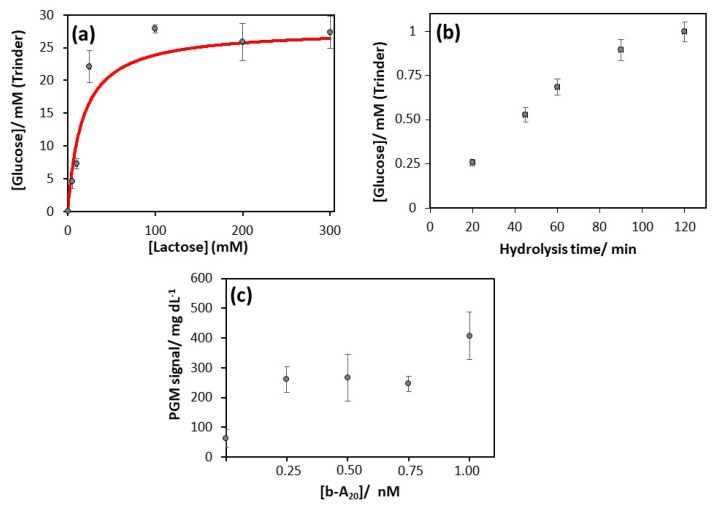
Studies of b-A_20_ detection by using β-galactosidase as glucose-generating enzyme. (**a**) Effect of lactose concentration on the analytical signal obtained by Trinder method. Red line represents the fit based on Michaelis–Menten model. (**b**) Effect of enzymatic hydrolysis time on the analytical signal obtained by Trinder method. (**c**) Detection of the model target b-A_20_ using the personal glucose meter under the optimized conditions.

Michaelis–Menten-type kinetic behavior was observed (Figure 2a) and, by fitting the experimental data to the corresponding equation, a Michaelis–Menten constant of (17 ± 6) mM was estimated. Given that the hydrolysis reaction rate should be independent of enzymatic substrate concentration, the suitable lactose concentration to be employed was that corresponding to β-galactosidase saturation and, accordingly, 100 mM of lactose was selected.

The enzymatic hydrolysis time also plays an important role in method sensitivity. To evaluate the effect of this parameter, the amount of glucose produced by enzymatic hydrolysis with β-galactosidase attached to magnetic particles modified with the sensing layer and hybridized with 2 µM of b-A_20_ was recorded by means of Trinder method. The obtained results (Figure 2b) show an increase in the generated glucose as the β-galactosidase reaction time increases, reaching a plateau after approximately 2 h. In order to attain the maximum sensitivity in the quantification of glucose, a hydrolysis time in the linear zone of maximum slope was chosen, specifically 60 min.

Once established, the optimized conditions for the β-galactosidase-catalyzed glucose generation (100 mM lactose at 30 °C and 1300 rpm for 60 min in 0.1 M phosphate buffer pH 7.1, containing 1 mM MgCl_2_), sensing layer-modified MBs were challenged to increasing concentrations of model target, b-A_20_, and the glucose produced was detected with the PGM. The signal recorded for amounts of b-A_20_ between 0.25 and 1 nM was significantly different from that for the blank (62 ± 29 mg glucose/dL in the absence of b-A_20_). However, the irreproducibility of the measurements did not allow quantification, thus resulting in a qualitative assay capable of detecting at least 250 pM b-A_20_ (Figure 2c). These assays were carried out on different days, using different aliquots of strep-β-Galac and even changing the enzyme conjugate batch. Trying to clarify the origin of the irreproducibility, enzymatic activity of different aliquots and batches of strep-β-Galac was checked with Trinder method, obtaining significant variations. This problem could be attributed to the commercial lyophilisate (enzyme conjugate not commonly used, synthesized on demand) or other uncontrolled factors affecting strep-β-Galac activity that make it difficult to obtain a PGM-based genoassay that is reliable enough.

### 3.2. Streptavidin-Alkaline Phosphatase

As a consequence of its high turnover number, a common enzyme label in the development of bioassays is alkaline phosphatase (ALP). It catalyzes the hydrolysis of a phosphomonoester, producing the corresponding molecule with free hydroxyl groups along with a new phosphate compound that, if the phosphate acceptor is water, will be an inorganic phosphate. ALP is commercially available conjugated to streptavidin (stoichiometry ≈ 2:1 streptavidin to ALP) at high purity, and it exhibits its maximum activity at pH values from 8 to 10.

Since PGM operates at physiological pH, a previous neutralization step of the solution resulting from ALP-catalyzed hydrolysis is required before glucose quantification. For this, 2 µL of 2 M HCl was added to 50 µL of hydrolysis solution before its application on the test strip. The use of a buffer solution of neutral pH instead of an acidic one could be a good alternative if PGM measurement is not performed immediately, although higher dilution would be required [22].

Among the phosphate containing compounds acting as glucose precursors, D-glucose-1-phosphate and D-glucose-6-phosphate can be commercially purchased. They are not the traditional ALP substrates used in bioassays; therefore, they are not expected to show the best performance. After testing both candidates, neither of them gave rise to a detectable signal on the glucose test strips, i.e., they did not contribute to the nonspecific PGM signal. Moreover, strep-ALP conjugate exhibited higher relative activity against D-glucose-1-phosphate (163 ± 4 mg dL^−1^ vs. 129 ± 11 mg dL^−1^ of glucose was recorded after 2.5 h of hydrolysis, when using 3 g/L of D-glucose-1-phosphate and D-glucose-6-phosphate, respectively), so it was selected for further studies. Nevertheless, these results depend on the source of the enzyme ALP [23].

**Figure 3:**
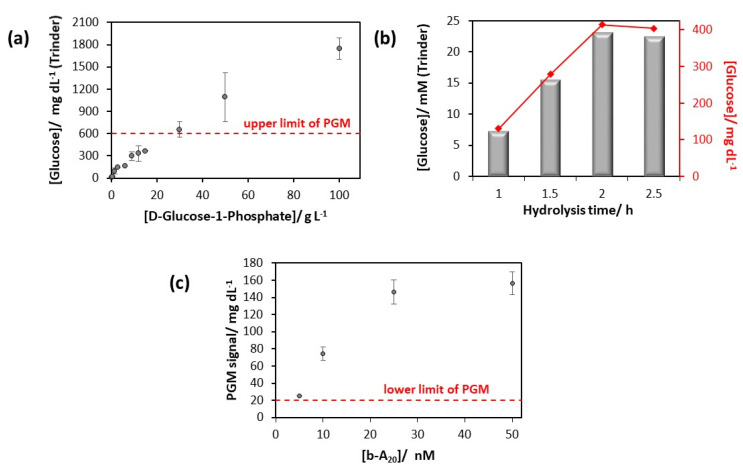
Studies of b-A_20_ detection by using ALP for catalytic glucose generation. (**a**) Effect of D-glucose-1-phosphate concentration on the analytical signal obtained by Trinder method. (**b**) Effect of enzymatic hydrolysis time on the analytical signal obtained by Trinder method. (**c**) Detection of the model target b-A_20_ using the PGM under the optimized conditions.

The conditions of the hydrolysis reaction catalyzed by ALP affect the performance of the future PGM-based genoassay. For this reason, the effect of D-glucose-1-phosphate concentration was first evaluated. To this aim, ALP specifically attached to MBs, which are modified with the sensing layer for the model target and hybridized with 2 µM of b-A_20_, was incubated with different concentrations of D-glucose-1-phosphate for 2.5 h, and the glucose produced was quantified by Trinder method. As illustrated in Figure 3a, no signal saturation was reached for D-glucose-1-phosphate concentrations between 0 and 100 g/L, thus meaning that the affinity of ALP toward D-glucose-1-phosphate is relatively poor. However, amounts of D-glucose-1-phosphate superior to 30 g/L produced glucose beyond the upper limit of the PGM; therefore, 30 g/L of D-glucose-1-phosphate was chosen for the next studies.

The reaction time of enzymatic hydrolysis was then optimized using magnetic microbeads modified in the same way as in the above studies (maximum amount of ALP conjugated to T_20_-A_20_ hybrids). As shown in Figure 3b, the glucose concentration recorded by Trinder method increased for the first two hours and then kept stable. Therefore, 2 h incubation time for ALP hydrolysis was selected for subsequent experiments.

Under optimized ALP catalysis conditions (30 g/L D-glucose-1-phosphate at 37 °C and 1300 rpm for 2 h in 0.1 M DEA pH 9.8, containing 1 mM MgCl_2_), the response of the PGM-based genoassay to b-A_20_ was evaluated. As can be seen in Figure 3c, the signal recorded with the glucose meter increases with the tested concentration of b-A_20_ in the range from 5 nM to 50 nM. Curiously, saturation of the sensing layer seems to be achieved at 50 nM of b-A_20_, even if the signal recorded (157 ± 14 mg/dL) is very far from the upper limit of the PGM (600 mg/dL). Taking into account the stoichiometry of the strep-ALP conjugate (2:1), it is quite likely that the number of ALP molecules incorporated per hybridization event is less than one [24]. Therefore, in order to improve the sensitivity of the PGM-based genoassay, a strategy based on the multivalent system biotin-streptavidin is envisaged. In particular, the presence of four biotin-binding sites per streptavidin molecule would allow for increasing the number of ALP molecules per hybrid formed on the MBs.

The approach comprises two new steps schematized in Figure 1a. First, free streptavidin binds with the biotinylated duplex immobilized onto the MBs, thereby occupying one out of four biotin binding sites in the protein structure. Subsequently, a BSA-biotin conjugate (stoichiometry: 8–16 moles of biotin per mole of albumin) is incorporated. As a result, the number of biotin moieties per hybridization event is significantly enhanced, leading to a greater number of attached strep-ALP conjugates. Strikingly, the PGM signal recorded for 0 nM (blank test) and 5 nM of b-A_20_ was found to be systematically lower than 20 mg/dL.

Although unexpected [25], the above results could be explained considering that streptavidin and BSA are very similar in size (60 kDa and 66 kDa, respectively). Thus, despite the multiple biotins conjugated to the same BSA molecule which, a priori, would favor a greater number of enzymes per b-A_20_ captured, steric hindrance could force the binding of a BSA-biotin conjugate with multiple duplexes because of the multivalence of the streptavidin, i.e., the same BSA-biotin conjugate serves as a bridge of several strep-b-A_20_-T_20_ hybrids. It would lead to fewer immobilized ALP molecules than hybridization events produced, and it would explain the decrease in signal when implementing this strategy. Furthermore, such negative effects on the signal could be worsened on magnetic beads with respect to planar surfaces, leading even to aggregation, as was the case here.

### 3.3. Antifluorescein-Fab Fragment-Alkaline Phosphatase

On account of the deleterious effect of the multivalent system biotin-streptavidin, a monovalent affinity reaction for ALP attachment was explored, namely antifluorescein Fab fragment–6-carboxyfluorescein (hereafter antiF-6FAM). Moreover, this approach was directly implemented for PCA3 detection by means of a sandwich-type bioassay (see Figure 1b) involving a 6FAM-tagged signaling probe (SP-6FAM) as well as an unmodified auxiliary probe (AuxP) listed in Table 1.

First of all, experimental conditions of D-glucose-1-phosphate hydrolysis catalyzed by ALP attached to the sandwich DNA onto MBs were revisited for optimum electrochemical detection. Glucose precursor concentration had to be increased from 30 to 100 g/L, while reaction time was kept at 2 h (data not shown).

**Figure 4:**
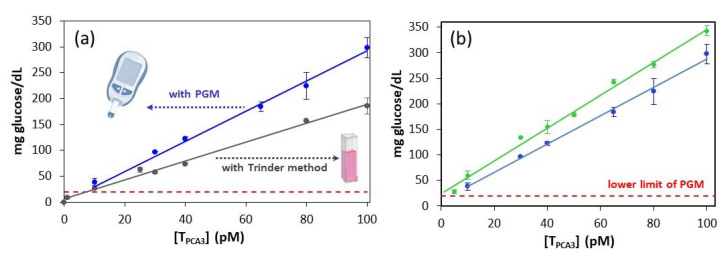
Calibration plots constructed for the determination of T_PCA3_. (**a**) One signaling probe grey: using colorimetric (Trinder method) and blue: using electrochemical (PGM) detection. (**b**) PGM readout: in green, two signaling probes; in blue, one signaling probe. Error bars were obtained through the detection of two parallel samples on two different days (four replicates in total). Equations of the calibration curves obtained in each case are presented in Table S1. Notice that the slope of the calibration curve obtained with the PGM-based method is significantly higher than that with Trinder method, thus resulting in a more sensitive genoassay.

Afterwards, the response of the PGM-based genoassay involving the antiF-6FAM labeling system toward the specific sequence of PCA3 was studied. As illustrated in Figure 4a, the concentration of glucose generated by ALP linearly increases with the concentration of T_PCA3_ in the range from 10 pM to 100 pM. This trend was observed with both spectrophotometric (Trinder method) and electrochemical (PGM) methodologies, although the latter exhibited higher sensitivity: (2.8 ± 0.1) and (1.81 ± 0.05) mg glucose dL^−1^ pM^−1^ with PGM and Trinder method, respectively (Table S1). The limit of quantification (LOQ) achieved with the PGM could be established close to 10 pM (5 femtomoles in 0.5 mL of sample), since the corresponding PGM signal was just above the lower limit of PGM (20 mg glucose/dL), while that for the blank was undetectable. These results show the convenience of the monovalent labeling strategy.

In an attempt to improve the detectability of the PGM-based genoassay, the auxiliary probe AuxP initially intended to assist in T_PCA3_ hybridization was modified in its 3′-end with a 6FAM moiety, thus becoming a second signaling probe and facilitating the incorporation of a second antiF-ALP conjugate per hybrid formed onto the MBs. The use of two signaling probes gave rise to a moderate enhancement of the method sensitivity (from 2.8 ± 0.1 to 3.3 ± 0.2 mg glucose dL^−1^ pM^−1^; Figure 4b and Table S1) and an experimental limit of quantification of 5 pM T_PCA3_. This modest improvement in the performance is quite reasonable considering that a variation of 1 mg/dL of glucose is required for recording a measurable change with the PGM.

Finally, the selectivity of the genoassay was evaluated against the interference I_PSA_, a DNA homolog of PSA mRNA, which is present in human urine. PGM readings corresponding to 100 pM of I_PSA_ were below the lower limit of the PGM (<20 mg glucose/dL), while for the same amount of T_PCA3_, a value of (343 ± 35) mg glucose/dL was recorded, thus evidencing excellent selectivity.

## 4. Discussion

In this work, instead of developing an entire platform for nucleic acid biomarker detection, we have focused on transforming the widely used personal glucose meter into an electrochemical transducer of hybridization events, by introducing alkaline phosphatase onto magnetic beads bearing the specific duplex for converting D-glucose-1-phosphate into D-glucose. Alkaline phosphatase has been previously reported in combination with PGM for measuring the activity of the enzyme galactose-1-phosphate uridyltransferase for galactosemia diagnosis [26]; however, to date, it has not been explored as a glucose-generating enzyme for nucleic acid quantification.

The selectivity of the developed assay was determined by Watson–Crick’s matching, while the sensitivity came from a signal amplification based on two enzymes: alkaline phosphatase (ALP), which catalyzes the production of glucose, and glucose oxidase (GOD), present in the glucose test strips, that transforms β-D-glucose into β-D-gluconolactone spontaneously hydrolyzed to gluconic acid. [Fe(CN)_6_]^3−^ then acts as a redox mediator transporting electrons from the enzyme to the surface of the working electrode. This redox reaction gives rise to the reduced form of the mediator, [Fe(CN)_6_]^4−^, and it is reoxidized at the electrode, generating an amperometric signal. As a result, a linear amplification is performed since one [Fe(CN)_6_]^4−^ is produced from each D-glucose-1-phosphate molecule.

This method exhibits a linear response at least between 5 and 100 pM of T_PCA3_. Concentrations greater than 100 pM have not been explored since the detection of nucleic acid biomarkers at nM levels is usually of little interest. The corresponding glucose signal varies between 24 and 350 mg/dL. The latter is far from the upper limit of the glucometer, where some PGM models lose their linear behavior [11]. Wider dynamic ranges have been described for nucleic acid quantification employing the PGM readout [27,28] but, considering the restricted detection range of the PGM (about one order of magnitude), the sensitivity of these genoassays tends to be lower, so a very small relative standard deviation is required for reliable quantification.

The quantification limit was found to be 5 pM when using two signaling probes that promote the labeling of each DNA duplex with two molecules of ALP. It should be pointed out that this is a value not estimated but experimentally determined, which favorably competes with those of previous works [14,28,29]. Lower values have also been reported [26,30,31,32] but the associated analytical methods entail numerous reagents and steps as well as longer times. Likewise, some examples involving isothermal nucleic acid amplification strategies, such as rolling circle amplification or RCA, onto magnetic beads have been also developed [33]. Nevertheless, marked aggregation, presumably due to the interaction between RCA products, was shown by Madaboosi and coworkers [34] when using different microscopy techniques, thus making difficult their tagging with the glucose-generating enzyme and in turn hampering glucose detection. They proposed to overcome the issue by working at low target concentrations, although a negative impact on the reproducibility of the results was evidenced.

Regarding the assay time, the affinity interaction between the target and its cognate receptor—that is, the hybridization event—mainly depends on the size of the first one and, consequently, it is conditioned by the intended application. Thus, short nucleic acids such as microRNAs tend to be detected faster [35] than long nucleic acid biomarkers as this is the case. However, the enzymatic reaction time suitable to generate enough glucose to be detected by the PGM is limited by the enzyme turnover number, which is temperature dependent, and it could be altered by enzyme functionalization. In this sense, the commercially available antiF-ALP conjugate presents a proper turnover number at normal human body temperature, compatible with simple equipment. A reduction in the volume in which the glucose is generated and measured has been proposed to shorten the enzymatic hydrolysis time [14], although this value should be enough to cover completely the sensing layer and to allow an appropriate agitation of the magnetic beads to obtain reproducible results. In this work, ≈10^7^ beads/mL in a total volume of 50 µL were employed.

Although current advances in the glucose monitoring field are aimed at developing minimal or non-invasive devices to reduce the issues related to traditional invasive methods, in parallel, scientific research efforts are focused on adapting commercially available glucose meters used for self-monitoring (i.e., personal glucose meter or PGM) for the determination of non-glucose targets. This approach would greatly expand the possibilities of point-of-care testing by taking advantage of decades of development of a device that has achieved approval for clinical analyses. Some changes in the current version of the transducer could help in this novel challenge. First, modification of the response range should be explored in order to enable the detection of lower glucose concentrations and therefore lower amounts of non-glucose target, while maintaining the upper threshold so as not to constrain the method sensitivity, vide supra. Second, redefining the units in such a way the recorded signal is not expressed as glucose concentration but in a more general and intuitive way could be considered. The third is related to selectivity. Glucose oxidation in the test strips can be conducted by either glucose oxidase or glucose dehydrogenases. Because of its higher selectivity toward glucose, glucose oxidase is preferable for diabetes control. However, with the purpose of quantifying non-glucose targets, less selective glucose dehydrogenases could be even better if a response is also generated toward the by-product of glucose generation (see the case of β-galactose resulting from the hydrolysis of lactose catalyzed by β-galactosidase). On the other hand, endogenous glucose present in human bodily fluids such as blood and, to a lesser extent, urine and tears could lead to non-negligible background signals. To circumvent this issue, different strategies have been proposed (performing two measurements, sample dilution, enzymatic removal) [11]. The use of magnetic beads as a solid support of the hybridization event can also help in this sense. Finally, although very convenient from a clinical point of view, especially considering self-monitoring, the safety mechanism that prevents the reuse of test strips is a limitation for research purposes and it would be interesting to redesign it as a function that could be disabled.

The ASSURED (affordable, sensitive, specific, user-friendly, rapid and robust, equipment-free and deliverable to end users) criteria proposed by the World Health Organization [36] serve as a reference for checking point-of-care devices. The methodology herein described for PCA3 detection based on a personal glucose meter matches reasonably well the ASSURED criteria. It is portable and inexpensive, presents good selectivity, and allows reliable quantification of 5 pM of PCA3. In this sense, even if upregulated in prostate cancer, the concentration of PCA3 in urine is scarce, and it may be present at a femtomolar level. Consequently, it forces the implementation of a previous preconcentration step that would be compatible with magnetic beads technology [37]. Likewise, to exclude any nonspecific variation, levels of PCA3 should be normalized with an internal control of stable expression, which demands the development of a parallel test for this latter. This could be achieved by using a second set of magnetic beads modified with suitable oligonucleotides for entrapping the endogenous control and subsequent measurement with a new glucose test strip. Regarding its use by untrained personnel (i.e., beyond health workers), although possible, this is not really demanded in this particular case. Thus, unlike diabetes that requires an immediate decision-making as a function of the glucose concentration throughout the day, cancer tends to progress slowly and its main problem is the lack of symptoms in the early stages when medical therapy can be beneficial for patients. This way, the methodology developed in this work, framed in the liquid biopsy diagnosis, could foster the massive screening of prostate cancer and therefore contribute to reducing suffering and death from prostate cancer.

## 5. Conclusions

A sandwich genoassay for quantification of PCA3, a urinary prostate cancer biomarker, has been developed by introducing alkaline phosphatase as a tracer, through a monovalent labeling system on a tagged hybrid formed onto magnetic beads. This enzyme is capable of producing glucose from D-glucose-1-phosphate, thus generating a signal on a personal glucose meter. This way, picomolar concentrations of a specific sequence of PCA3 were amplified into millimolar concentrations of glucose, that can be detected with the commercially available PGM, and the recorded signal was directly proportional to the concentration of the biomarker. The use of two signaling probes allowed the incorporation of two ALP molecules per analyte, resulting in a LOQ of 5 pM. Likewise, excellent selectivity against a DNA homolog of PSA mRNA, also present in urine of prostate cancer patients, was achieved. This methodology has the potential to be applied to the portable and quantitative detection of other nucleic acid biomarkers by proper design of probes.

## Figures and Tables

**Table 1:** Oligonucleotides used throughout the work.

Name	Role	Sequence (from 5′ to 3′)
b-T_20_	Biotinylated capture probe	Biotin-TTTTTTTTTTTTTTTTTTTT
b-A_20_	Model target	Biotin-AAAAAAAAAAAAAAAAAAAA
b-CP	Biotinylated PCA3 capture probe	Biotin-(T)_6_-AATTCTGGCTTCTGCTGAGAAT
SP-6FAM	6FAM-Signaling probe	AGACCTAATGCAAGT-6FAM
AuxP	Auxiliary probe	ACCGCCTGATGCACAG
AuxP6FAM	6FAM-Auxiliary probe	ACCGCCTGATGCACAG-6FAM
T_PCA3_	Target PCA3	AAGCAAAATACTTGCATTAGGTCTCAGCTGGGGCTGTGCATCA GGCGGTTTGAGAAATATTCAATTCTCAGCAGAAGCCAGAATT
I_PSA_	Interference PSA	GGTCTTCCTTTGGCATGGGATGGGGATGAAGTAAGGAGAGGGACTGGACCCCCTGGAAGCTGATTC

## Supplementary Materials

The following are available online at https://www.mdpi.com/1424-8220/20/19/5514/s1. The initial adjustments of PGM and Table S1: Analytical performance of Trinder and PGM-based genoassay.
