# Supplementary material for: New Uses for the Personal Glucose Meter: Detection of Nucleic Acid Biomarkers for Prostate Cancer Screening
Source: Sensors (Basel). 2020 Sep 26;20(19):5514. doi: 10.3390/s20195514 (PMC7583019; doi:10.3390/s20195514)
Supplement: Supplementary file 1 [file sensors-20-05514-s001.pdf]

Supplementary Material

New Uses for the Personal Glucose Meter: Detection of Nucleic Acid Biomarkers for Prostate Cancer Screening

Clara Abardía-Serrano [1,2], Rebeca Miranda-Castro [1,2], N. de-los-Santos-Álvarez [1,2] and María Jesús Lobo-Castañón [1,2,*]

[1] Departamento de Química Física y Analítica, Universidad de Oviedo, Av. Julián Clavería 8, 33006 Oviedo, Spain; abardiaclara@uniovi.es (C.A.-S.); mirandarebeca@uniovi.es (R.M.-C.); santosnoemi@uniovi.es (N.d.-l.-S.-Á.)

[2] Instituto de Investigación Sanitaria del Principado de Asturias, Avenida de Roma, 33011 Oviedo, Spain

* Correspondence: mjlc@uniovi.es; Tel.: +34-98-510-6235

1. Initial adjustments of PGM

The measurement range with GlucoMen® Areo Sensor test strips is from 20 to 600 mg glucose/ dL human blood. However, in this work it is intended to detect glucose in aqueous solutions whose viscosity is significantly lower than that of the blood. Therefore, optimization of the sample volume in contact with the glucose test strip is required. For this purpose, a solution of 500 mg glucose/ dL water was employed, and different attempts were performed by using different test strips (notice that the glucometer has a safety mechanism that prevents the reuse of test strips). The optimal volume for the measurement in water turned out to be 30 μL, which guarantees that the sample reaches the electrodes by capillarity.

Then, increasing concentrations of glucose in water were evaluated with the PGM, finding a good correlation between the actual glucose concentration and that detected by the PGM in water, when using the optimized sample volume. Specifically, the slope of the resulting representation (PGM signal *vs.* glucose concentration in aqueous sample) was 0.97±0.04, very close to one.

2. Table S1

Table S1. Analytical performance of Trinder and PGM-based genoassays.

Method	N_{SP}	Calibration curve	Dynamic range	LOQ
Trinder	1	[Glucose] (mg dL^{-1}) = (1.81±0.05) [T_{PCA3}](pM)+ (7±3)<br>R = 0.998	1-100 pM	16 pM*
PGM	1	[Glucose] (mg dL^{-1}) = (2.8±0.1) [T_{PCA3}](pM)+ (10±6)<br>R = 0.998	10-100 pM	10 pM**
	2	[Glucose] (mg dL^{-1}) = (3.3±0.2) [T_{PCA3}](pM)+ (24±7)<br>R =0.996	5-100 pM	5 pM**

N_{SP}: number of signaling probes

(*) calculated as ten times the standard deviation of the intercept divided by the slope.

(**) experimentally determined.
